# A stepwise activation model for the insulin receptor

**DOI:** 10.1038/s12276-023-01101-1

**Published:** 2023-10-02

**Authors:** Na-Oh Yunn, Junhong Kim, Sung Ho Ryu, Yunje Cho

**Affiliations:** 1 Postech Biotech Center, Pohang University of Science and Technology (POSTECH), Pohang, 37673 Republic of Korea; 2 Department of Life Sciences, Pohang University of Science and Technology (POSTECH), Pohang, 37673 Republic of Korea; 3 Department of Biomedical Science and Engineering, Pohang University of Science and Technology (POSTECH), Pohang, 37673 Republic of Korea

**Keywords:** Chemokines, Insulin signalling, Metabolomics

## Abstract

The binding of insulin to the insulin receptor (IR) triggers a cascade of receptor conformational changes and autophosphorylation, leading to the activation of metabolic and mitogenic pathways. Recent advances in the structural and functional analyses of IR have revealed the conformations of the extracellular domains of the IR in inactive and fully activated states. However, the early activation mechanisms of this receptor remain poorly understood. The structures of partially activated IR in complex with aptamers provide clues for understanding the initial activation mechanism. In this review, we discuss the structural and functional features of IR complexed with various ligands and propose a model to explain the sequential activation mechanism. Moreover, we discuss the structures of IR complexed with biased agonists that selectively activate metabolic pathways and provide insights into the design of selective agonists and their clinical implications.

## Decoding the Sequential Activation of Insulin Receptor

New insights into insulin receptor activation mechanisms reveal a stepwise process, providing a basis for designing functionally selective insulin receptor agonists. Cryo-EM structures of the insulin receptor in various states, from inactive to fully active, have been analyzed, and a model for insulin-induced activation has been proposed. This model suggests that insulin binding leads to transient intermediate states before reaching the fully active conformation. These intermediate states display functional selectivity biased toward metabolic effects rather than mitogenic signaling. Understanding these activation mechanisms may aid in the development of novel therapeutic strategies for diabetes and other insulin-related disorders.

## Introduction

Insulin binding to the insulin receptor (IR) triggers sequential conformational changes and autophosphorylation of the receptor, followed by activation of a kinase signaling cascade that plays essential roles in a wide variety of biological processes^1,2^. IR is a critical regulator of glucose and lipid metabolism. Its metabolic effects, including glucose uptake and lipid synthesis, are primarily regulated by the phosphoinositide 3-kinase (PI3K)/AKT pathway. In muscle and adipose tissues, IR signaling leads to GLUT4 translocation, increasing blood glucose uptake into these tissues. In the liver, insulin increases glycogen and lipid synthesis and decreases gluconeogenesis. IR signaling promotes cell growth, proliferation, and development. Although the PI3K/AKT pathway is involved, the mitogen-activated protein kinase (MAPK) pathway is the primary mediator of the mitogenic effects of insulin^3,4^.

IR signaling dysfunction, caused by pancreatic beta cell destruction or insulin resistance, chronically elevates blood glucose levels, resulting in the metabolic disease diabetes mellitus^5^. Patients with diabetes suffer from a variety of complications, including nephropathy, retinopathy, peripheral vascular diseases, cardiovascular diseases, atherosclerosis, and stroke^6,7^. Mounting evidence indicates that diabetes mellitus increases the risk of cancer progression and mortality^8^. As blood glucose levels must be lowered to treat diabetic complications, IR is an important target for diabetes treatment. However, the detailed mechanism underlying insulin-binding-induced IR activation remains to be elucidated.

Recently, advances in cryogenic electron microscopy (cryo-EM) have significantly expanded the range of identified IR structures in different states, improving our understanding of the IR activation mechanism. Studies on full-length IR complexed with insulin, peptides, and aptamers have revealed various extracellular domain (ECD) structures of ligand-bound IR^9–20^. Despite breakthroughs in the structural analysis of insulin-bound IR, the current understanding of the IR activation mechanism is limited. First, how insulin accesses the apo IR binding sites and induces structural transitions during its activation remains unclear^21^. Characterization of such transient intermediate structures is challenging because of their intrinsic instability. Second, structural studies have been largely limited to the IR ECD despite full-length IR being used for structure analyses. Thus, the coordination of ECD conformational changes and cytoplasmic kinase domain autophosphorylation remains unresolved. Third, the current understanding of how agonists that induce functional selectivity are biased toward certain metabolic effects is limited^22–25^. Recently, structures of aptamer-trapped IR have been reported and provide clues to the IR activation mechanism, as their structures likely represent intermediate IR states^9^. IR interactions with these agonists suggest that IR activation may involve its transition through different conformational states and subsequent autophosphorylation events.

In this review, we describe IR structures and proposes a model to explain the early-stage IR activation mechanism. We focus mainly on the structural transition from an apo to a fully active single-insulin-bound IR under physiological conditions because several excellent reviews have discussed multiple-insulin-bound IR structures^26,27^. We further address the biological and structural backgrounds of selective agonist-bound IRs and provide insights into the design of selective agonists and their clinical implications.

## IR domain organization

**Fig. 1:**
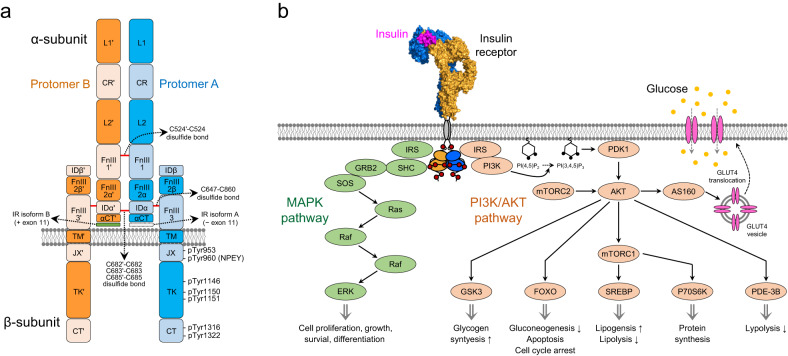
Domain architecture and signal pathway of IR. **a** IR domain architecture composed of two α-subunits and two β-subunits. Protomer A and B are colored blue and orange, respectively. A prime (′) symbol represents the opposite protomer element. Red lines: disulfide bonds. **b** Two major signaling pathways initiated from insulin-induced IR activation. Insulin binding to IR induces autophosphorylation on multiple tyrosine residues, which recruits and phosphorylates IRS and SHC. IRS proteins initiate the PI3K–AKT pathway by activating PI3K. SHC proteins mainly initiate the Grb2-SosRas-Raf-ERK cascade (MAPK pathway).

IR belongs to a class of receptor tyrosine kinases (RTKs) that comprises 58 receptors in humans^28^. Similar to other RTKs, the IR extracellular (ectodomain) and cytoplasmic domains are linked by a single transmembrane helix. However, IR family members, including IR and insulin-like growth factor-1 receptor (IGF-1R), have structural characteristics that differ from those of other RTKs (Fig. 1a). First, the receptor precursor is proteolytically separated into α- and β-subunits, which are stably linked by a disulfide bond. The IR ECD consists of the whole α-subunit and part of the β-subunit; the other part of the β-subunit is included in the single transmembrane helix and intracellular region. Second, the IR holoreceptor is a disulfide-linked (αβ)_2_ homodimer, in contrast to other RTKs that undergo dynamic conversion between monomers and dimers. Owing to the disulfide bonds between the two α-subunits, IR exists as a covalently linked dimer on the plasma membrane.

The IR α-subunit functions as a ligand-binding site and is composed of a leucine-rich repeat (L1), a cysteine-rich region (CR), a second leucine-rich repeat (L2), two fibronectin type III domains (FnIII-1 and FnIII-2a), an insert domain α (IDα), and an α-helical C-terminal domain (αCT; Fig. 1a). The IR β-subunit includes extracellular insert domain β (IDβ) and fibronectin type III domains (FnIII-2b and FnIII-3). The transmembrane (TM) helix, intracellular juxtamembrane (JM), tyrosine kinase (TK), and C-terminal tail (CT) domains belong to the β-subunit. The alternative splicing of Exon 11 (12 amino acids) in the IR gene transcript determines the αCT domain length, giving rise to two IR isoforms, A and B. However, only IR isoform B contains the Exon 11.

## IR activation

Insulin binds to IR in peripheral tissues, initiating receptor activation followed by intracellular signaling cascades^29^. The first step in IR activation is the autophosphorylation of intracellular tyrosine residues in the JM domain, kinase activation loop, and CT domain^30^. Phosphorylation of three tyrosine residues (Tyr1146, Tyr1150, and Tyr1151, based on IR isoform A numbering) located in the kinase activation loop plays a crucial role in kinase activity regulation. Biochemical analyses have suggested that these sites are sequentially phosphorylated in the following order: Tyr1150, Tyr1148, and Tyr1151^31,32^. In inactive IR, an unphosphorylated activation loop is inserted into the catalytic site of the kinase, inhibiting kinase activity by blocking the substrate and ATP binding to this site^33^. After insulin-binding-induced initial IR phosphorylation, the phosphorylated activation loop is released from the catalytic site, which increases IR kinase activity by allowing substrate and ATP interactions with the kinase^34^. Insulin binding also induces IR kinase-mediated phosphorylation of four tyrosine residues located in the JM (Tyr953 and Tyr960) and CT domain (Tyr1316 and Tyr1322)^35–37^. Phosphorylated Tyr960 (pY960) functions as an NPXY motif, a docking site for the phosphotyrosine-binding domain of IR substrate (IRS) and Src homology 2 domain-containing transforming protein (SHC)^38,39^. IRS and SHC recruitment to the JM domain leads to IR kinase-mediated IRS-1 and SHC phosphorylation, which in turn promotes various intracellular signaling events (Fig. 1b). Several reports suggest that phosphorylated Tyr1316 (pY1316) and Tyr1322 (pY1322) are involved in IR kinase activity and kinase–adaptor protein interactions; however, their specific roles in IR activation and signaling remain unclear^40–42^.

IR activation mediates two major signaling pathways: the PI3K/AKT and MAPK pathways^29^. The insulin-activated PI3K/AKT pathway is responsible for the majority of the metabolic effects mediated by insulin in peripheral tissues^43^. IRS proteins interact with the p85 regulatory subunit of PI3K, activating PI3K to generate the second messenger phosphatidylinositol-3,4,5-trisphosphate (PIP3). PIP3 recruits phosphoinositide-dependent protein kinase-1 (PDK1) to the plasma membrane and activates AKT serine/threonine kinase, which in turn phosphorylates various proteins, leading to glucose uptake by glucose transporter type 4, glycogen synthesis by GSK3, protein and fat synthesis by mTOR, and gene expression by the Forkhead Box O transcription factor. The MAPK pathway, which includes the Grb2-Sos-Ras-Raf-ERK cascades, is crucial for regulating the mitogenic effects of insulin on cell proliferation, growth, and differentiation^44^. Although the MAPK pathway is initiated by both SHC and IRS, SHC is the principal initiator. ERK, activated by insulin stimulation, mediates the phosphorylation of nuclear translocated cytosolic proteins and functions as a transcription factor.

## Inactive state of the IR

**Fig. 2:**
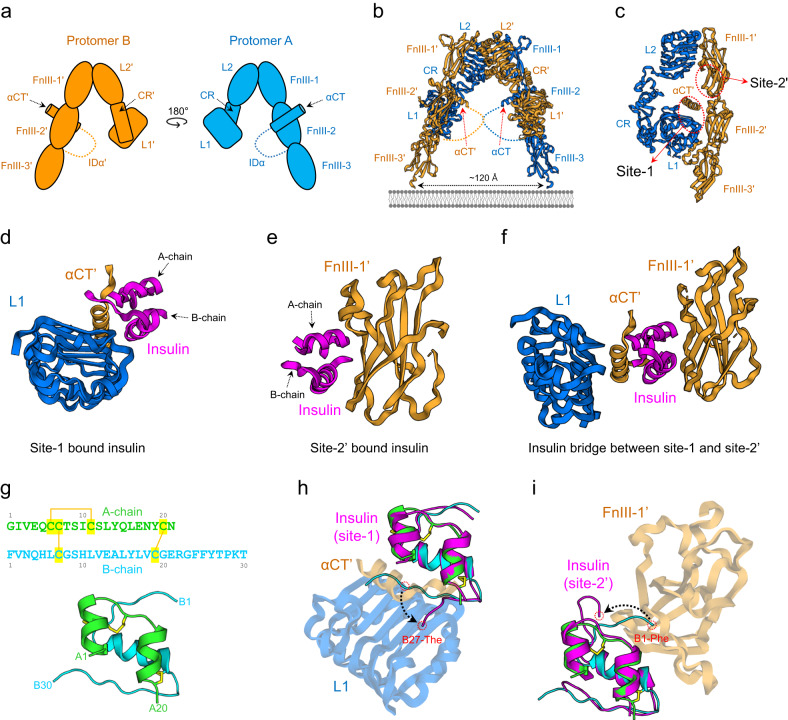
Inactive conformation and two insulin binding sites of IR. **a** Schematic diagram of IR based on the apo (unliganded) structure. Protomer A and B are colored blue and orange, respectively. **b** The X-ray crystal structure of apo IR (PDB ID: 4ZXB). Two hook-like protomers form a symmetric Λ-shaped dimer in an antiparallel manner. **c** Orthogonal view of one-half of the Λ-shaped IR homodimer. Two insulin binding sites, site-1 and site-2’, are shown in red dotted circles. **d** Close-up view of insulin-bound site-1 in the Γ-shaped IR structure (PDB ID: 7YQ3). **e** Close-up view of insulin-bound site-2’ in the T-shaped IR (PDB: 6PXV). **f** Close-up view of the insulin bridge between site-1 and site-2’ in the two-insulin-bound tilted T-shaped structure (PDB ID: 7STK). **g** Amino acid sequence and structure (PDB ID: 1MSO) of human insulin. The A-chain and B-chain are colored green and cyan, respectively. Yellow lines indicate disulfide bonds. **h** Superimposition of free insulin (PDB ID: 1MSO; green and cyan) and insulin-bound site-1 (PDB ID: 6VEP; pink). **i** Superimposition of free insulin (PDB ID: 1MSO; green and cyan) and insulin-bound site-2’ (PDB ID: 6SOF; pink).

The crystal structure of the IR ectodomain (PDB ID: 4ZXB) revealed that the extracellular (αβ)_2_ homodimer forms an Λ (inverted V)-shaped structure in the apo state^45,46^. In the crystal structure, this shape is stabilized by two antibodies (Fig. 2a, b). The extracellular αβ monomer forms a hook-like structure. The L1, CR, and L2 domains form the head of the hook, and the linearly arranged FnIII-1, FnIII-2, and FnIII-3 domains form the stalk. Two αβ monomers are combined to form a symmetric Λ-shaped dimer in the antiparallel orientation, and one protomer interacts with the other via five interfaces.

In each half of the Λ-shaped homodimer, the L1 and L2 domains of one protomer interact with the FnIII-1′, aCT′, and FnIII-2′ domains of the other half of the dimer (′ represents opposite protomer elements) (Fig. 2b left). At the top, the FnIII-1’ and L2 domains, which are positioned in the front and back, respectively, constitute the first interface via hydrophobic and ion-pair interactions. The L2’ and L2 domains, which cross the center of the Λ-shaped homodimer, form the second interface via ion pairs, H-bonds, and phi–phi stacking interactions (Fig. 2b left). In the middle, three interfaces (L1–FnIII-2′, L1–αCT′, and L1–ID′) further stabilize the structure (Fig. 2b right). At the L1–FnIII-2′ interface, residues in the C-terminal side of the L1 β-strands form hydrophobic interactions with residues in the FnIII-2′ strand. At the L1–αCT′ interface, αCT′ residues form hydrophobic contacts with the residues in the β-sheet of L1. The C-terminal end of L1 forms an interface with ID′ via hydrophobic interactions and ion pairing.

Despite the extensive interactions between the two protomers, the Λ-shaped IR structure may be unstable and highly flexible considering that two antibodies were required for crystallization. Recently, three additional Λ-shaped structures were identified using cryo-EM. These structures were also detected in the presence of a peptide agonist (S597 component 2; 8DTM), modified insulin (7SL1), and an insulin mutant lacking site-1 binding (IRPA-3 partial agonist; 7MD4)^15,17,18^. In these structures, all ligands bind to the outer surface of FnIII-1 at the same binding site of the antibody used for crystallization studies^45,46^.

In the Λ-shaped conformation, the membrane-proximal ends of FnIII-3 and FnIII-3′ are ~120 Å apart. As the high-resolution structure of full-length IR-containing intracellular kinases is not available, the structural arrangement of intracellular kinases remains unknown. However, assuming that the kinase is located close to the TM domain, the distance between FnIII-3 and FnIII-3 termini at the membrane is sufficient to prevent *trans*-autophosphorylation between kinases.

## Insulin-binding sites

Biochemical and structural studies on insulin binding to its receptor have proposed that each IR monomer harbors two different insulin-binding regions, a primary (site-1) and secondary (site-2) site (Fig. 2c). Site-1 is composed of the L1 and αCT′ domains, and the interaction between these domains is critical for insulin binding and IR activation (Fig. 2d)^10,21^. Alanine scanning analysis of L1 revealed that the residues on the L1 β-sheet surface are involved in the insulin–IR interaction and that many of these residues are in contact with αCT′^47–49^. This suggests that both protomers participate in the formation of site-1, and the L1 and αCT′ tandem element complex serves as a platform for insulin binding.

Site-2′ is located on the FnIII-1 β-sheet surface, and insulin binds to it either in coordination with site-1 or independently (Figs. 2e and 2f)^12,13,15,16^. In the holo-IR (IR dimer), the two protomers are arranged in a symmetric antiparallel direction. Site-1 and site-2′ are located on one side, while site-1′ and site-2 are positioned on the other. Owing to the antiparallel dimer formation of IR, site-1 and site-2′ (site-1′ and site-2) on one side face each other. The insulin binding affinities for site-1 and site-2 differ markedly; insulin binds to site-1 with high affinity (*K*_*d*_ 10–30 nM) but with much weaker affinity (*K*_*d*_ ~ 400 nM) for site-2^21,50,51^. Therefore, its binding to site-2′ is observed only when 2 to 4 insulin molecules bind to a single IR dimer, which occurs at excessive insulin concentrations (>100 nM) above physiological conditions (~pM).

## Conformational changes of the insulin bound to site-1 or site-2’

Fully mature insulin consists of an A-chain (21 residues, A1–A21) and a B-chain (30 residues, B1–B30) linked by two disulfide bonds (Fig. 2g). Two short α-helices of the A-chain are arranged in parallel and have an inner disulfide bond between A6-Cys and A10-Cys. The B-chain consists of an N-terminal segment (B1–B6), central α-helix (B9–B19), and C-terminal β-strand (B24–B28). When insulin binds to site-1, the C-terminal segment (B24–B30) of the insulin B-chain is folded out from the central α-helix, and three hydrophobic residues (B24-Phe, B25-Phe, and B26-Tyr) engage with the L1 and αCT′ tandem element^52^ (Fig. 2h). These three hydrophobic residues play a critical role in normal insulin action^53^. When insulin interacts with site-2’, the insulin B-chain N-terminal segment (B1–B5) also bends away from the FnIII-1 β-sheet surface (Fig. 2i)^13^. The first three residues (B1–B3) seem to be in close proximity to site-2’; however, their effects on insulin activity are insignificant^53^. Structural details of insulin alone and IR-bound insulin have been extensively described in another excellent review^26^.

## Single-insulin-bound fully active state of the IR

**Fig. 3:**
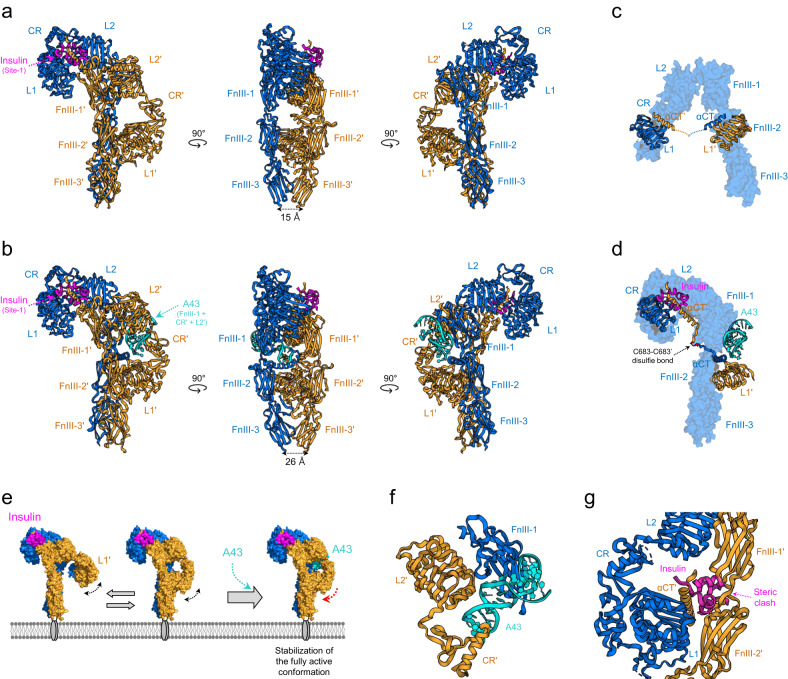
Fully active Г-shaped IR bound to a single insulin molecule. **a** Cryo-EM structure of the IR extracellular region with the leucine-zipper domain fused to the C-terminus (6HN5 and 6HN4). **b** Cryo-EM structure of full-length IR bound to one insulin molecule and one A43 aptamer (PDB ID: 7YQ3). The A43 aptamer fits in a pocket comprising FnIII-1, CR′, and L2′ on the insulin-free side. **c** Close-up view of αCT and αCT′ interacting with L1’ and L1, respectively, in the apo IR structure (PDB ID: 4ZXB). The crosslink between insert domains is missing in the structure. **d** Close-up view of the αCT-αCT′ bridge cross-linked by the C683-C683’ disulfide bond in the A43-bound Γ-shaped IR structure. **e** A model for the insulin-enhancing activity of the A43 aptamer. When a single insulin molecule binds to IR leads to the Γ-shaped conformation, the position of the opposite L1’ may become unstable. Because of the αCT-αCT′ bridge, the structural instability of L1’ can interfere with insulin binding to site-1 (L1’ and αCT′) by increasing steric tension between L1 and L1′. A43 binding to IR can stabilize the Γ-shaped conformation by fixing the position of L1’, which enhances insulin-induced IR phosphorylation by stabilizing insulin binding to site-1. **f** Close-up view of A43 in a pocket comprising FnIII-1, CR′, and L2′ in Fig. 3b. **g** Superposition of insulin bound to site-1 in the Γ-shaped IR structure onto the Λ-shaped apo IR by aligning the L1 domain to demonstrate the steric clash of insulin. Steric clash of insulin (pink) with FnIII-1’ and FnIII-2’ (orange) is evident.

Cryo-EM of single-insulin-bound IR revealed that insulin binding to site-1 leads to an asymmetric Γ-shaped IR structure (Fig. 3a, b)^9,10^. This is the first structural evidence showing that insulin binding changes the conformation of the IR dimer. The first Γ-shaped structure was observed in a soluble IR with the leucine-zipper domain fused to the C-terminus of IR-ECD (IRΔβ-zip) (Fig. 3a)^10^. The single-insulin-bound Γ-shaped IR represents the fully activated conformation. Conclusions drawn with this model are supported by the structures of the IGF-1R–IGF-1 complex, a small population of particles in a low concentration of insulin-treated IR, and the A43 aptamer (a positive allosteric modulator)-bound IR (Fig. 3b)^9,16,54^. In the Γ-shaped structure, L1, CR, L2, and FnIII-1 form an upshifted head in one protomer (blue) and a downshifted head in the opposite protomer (orange); the stalks comprise FnIII-1, FnIII-2, and FnIII-3 in both protomers.

In this activated IR, insulin simultaneously binds to L1 and αCT′ at site-1, while the opposite side is insulin free. L1 and αCT′ at site-1 are lifted up toward FnIII-1′ compared to that in apo IR. The hydrophobic residues of the insulin B-chain (V12 and F24) are directed toward the hydrophobic interface between L1 and αCT′ (L37, F39, F64, and F96 of L1 and L709, V713, and F714 of αCT′) and stabilize the interface. Although residues that participate in the hydrophobic interaction are almost the same as those that interact in the apo state, these interactions slightly change the angle between L1 and αCT′^21^. A phi–phi interaction (between F39 of L1 and Y16 of insulin) and a polar interaction (between R19 of L1 and Y26 of insulin) provide additional binding force between L1 and the insulin B chain^10,55^. Moreover, insulin (chain A) is located close to the upper loop of FnIII-1′. The upper loop of FnIII-1′ is located close to a disulfide bond (A7-Cys and B7-Cys), B5-His and B10-His of insulin, and might act as an additional interface (site-1b)^10,12^.

L1′ on the insulin-free side of the asymmetric dimer is subsequently translocated toward the FnIII-2 and FnIII-2′ stalks compared to its position in the apo IR dimer (Figs. 2b and 3a, b). The rearrangement of the heads on each side brings the two stalks in close proximity, markedly reducing the distance between the membrane-proximal ends of FnIII-3 and FnIII-3′ from 120 Å to 15–26 Å. A key feature is the conformational change and associated movement of the αCT–αCT′ bridge linked by the C683–C683’ disulfide bond; the αCT with an N-terminal four-turn helix in the apo IR is extended to form a seven-turn helix and a loop (Fig. 3c, d). The upshift of one αCT forces the opposite αCT′ downward as their ends are connected by a disulfide bond. Thus, the shift of the two FnIII-3 ends brings transmembrane helices and cytoplasmic kinase domains into close proximity, potentially allowing *trans*-autophosphorylation and activating downstream signaling cascades.

The A43 aptamer, a single-stranded DNA aptamer consisting of 31 nucleic acids, functions as a positive allosteric modulator that increases insulin binding to the IR and subsequent IR phosphorylation^56^. The A43-bound IR structure is almost identical to the Γ-shaped structure (Fig. 3b)^9^. Although the dissociation constant (*K*_d_) of a single insulin molecule binding to the IR is in the subnanomolar range, it is difficult to obtain a stable Γ-shaped structure without antibody or aptamer binding^9,10^. This suggests that the conformation of the insulin-free side is heterogeneous. Furthermore, stabilization of the Γ-shaped structure enhances insulin-induced IR phosphorylation (Fig. 3e).

In the IR–A43 complex, the A43 aptamer fits in a pocket comprising FnIII-1, CR′, and L2′ on the insulin-free side (Fig. 3f). The loop of A43 interacts with CR′ (R271), and one side of the phosphate backbone forms hydrogen bonds with L2′ (S323 and T325). A43 also binds to the outside of FnIII-1 (strands A, B, and E), similar to ligands that bind to FnIII-1 (site-2). The phosphate backbone, or the internal base of A43, forms an ion pair with arginine residues (R488 and R554) or aromatic residues (Y477 and W551) that participate in ring stacking with bases. The main interaction is formed between hydrophobic residues (L486 and L552) and the naphthyl group of the modified base in A43^9,56^.

In the Γ-shaped active structure, bound insulin forms a stable complex with L1 and αCT′. However, when the insulin-binding module in the Γ-shaped structure was superimposed onto L1 of the inactive Λ-shaped structure, it was clear that insulin binding can cause substantial steric clash with nearby FnIII-2′ in the apo state (Fig. 3g)^21^. This finding suggests that insulin cannot directly access the primary binding site in the Λ-shaped structure, and the initial insulin binding to apo IR requires the translocation of FnIII-2′ away from L1. However, the mechanism underlying this transition remains unclear. Capturing the transient states representing the initial insulin binding to the IR and transition to subsequent IR conformational changes is challenging owing to the intrinsic instability of apo IR.

## A62 aptamer-trapped monophosphorylated IR

**Fig. 4:**
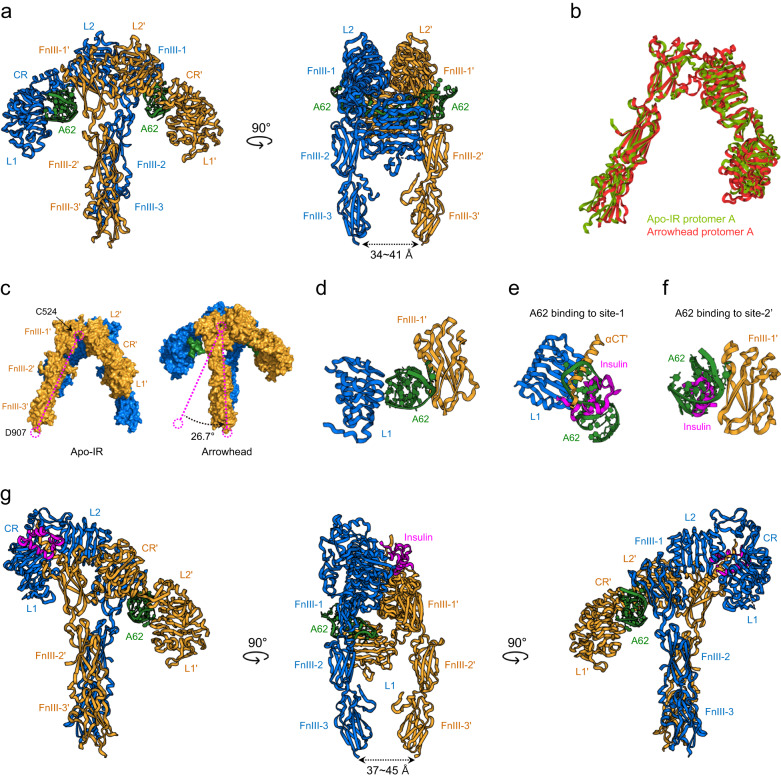
A62 aptamer-trapped monophosphorylated IR. **a** The cryo-EM structure of full-length IR bound to two A62 aptamers (PDB ID: 7YQ6). The A62 aptamers are green. **b** Superposition of the protomers of the Λ-shaped apo structure (green, Fig. 2b) and arrowhead-shaped IR structure (red, Fig. 4a). **c** Comparison of the Λ-shaped apo IR structures (left) and two A62-bound IR structures (right). Protomer rotation by 26.7° with respect to Cys524 converts the Λ-shaped IR structure to the arrowhead-shaped IR structure. **d** Close-up view of the A62 bridge established between L1 and FnIII-1′ in arrowhead-shaped IR. **e** Superimposition of insulin-bound site-1 (PDB ID: 7YQ3) and A62-bound L1 in arrowhead-shaped IR. **f** Superimposition of insulin-bound site-2’ (PDB ID: 6PXV) and A62-bound FnIII-1’ in the arrowhead-shaped IR. The A62 binding site overlaps with insulin-binding site-1 and site-2’. **g** The cryo-EM structure of the tilted T-shaped IR complexed with one A62 aptamer and one insulin molecule (PDB ID: 7YQ5) in three different views.

A62, a biased agonist aptamer, selectively activates the PI3K/AKT pathway and metabolic effects of IR^23^. A62 comprises 25 nucleotides, including modified nucleotides. A key feature of A62 is its selective mono-phosphorylation of Tyr1150 (m-pY1150) in the IR, which is the first phosphorylated site in the kinase activation loop. Therefore, it is reasonable to speculate that the A62-bound IR conformation represents an intermediate state between the inactive apo Λ-shaped and the fully active Г-shaped IR. A62-complexed IR, determined at an average resolution of 4.2 Å, forms a symmetric arrowhead-shaped structure in which two A62 aptamers bind to each side of the receptor dimer (Fig. 4a)^9^. The head consists of L1, CR, L2, and part of FnIII-1 in both protomers, while the stalk comprises parallel FnIII-2 and FnIII-3. In the head, L1, CR, and L2 are linearly arranged in an extended conformation, similar to the crystal structure of partial IR domains comprising L1, CR, L2, FnIII-1, and αCT, in which insulin binds to L1 and αCT (PDB ID: 5KQV)^21^.

The dimer interface of the arrowhead-shaped IR structure is formed by FnIII-1 and L2′, without extensive contact. The structure of the entire protomer of the IR–A62 complex is identical to that of apo IR (Fig. 4b); the rigid body of each protomer in the Λ-shaped IR rotates 26.7° toward each other with respect to Cys524 (FnIII-1), which leads to the detachment of L1 from FnIII-2′, resulting in the arrowhead-shaped structure (Fig. 4c). As the Λ-shaped apo IR is flexible, we speculate that the ligand-free IR undergoes a dynamic transition between the Λ- and arrowhead-shaped conformations and that A62 captures the two protomers in a specific arrangement in the arrowhead-shaped conformation (i.e., A62 stabilizes this specific arrangement of the arrowhead form by interacting with head and stalk elements). A key difference between the A62-bound arrowhead- and Γ-shaped structures is the distance between the two membrane proximal ends of FnIII-3 and FnIII-3′. Although FnIII-3 and FnIII-3′ appear to be parallel, similar to those in the Γ-shaped structure from the front view, the distance between them is 34–41 Å.

The A62 aptamer simultaneously binds to L1 and FnIII-1’ (Fig. 4d). One face of A62 interacts with L1′ through the A62 stem, the backbone phosphates form ion pairs with two arginine residues (R14 and R65) in L1′, and the A62 naphthyl group forms hydrophobic interactions with F64. Because A62 and αCT competitively interact with L1′, αCT is dissociated from L1′, and not visible in the IR–A62 complex (Fig. 4e). On the opposite face of A62, backbone phosphates form ion pairs with three arginine residues (R479, R488, and R554) in FnIII-1′. Several A62 bases form ring-stacking interactions with Y477 in FnIII-1. A62 covers site-2, interacting with R488 and R554, which also bind to insulin (Fig. 4f; details of these interactions are described in refs. ^9,12,13^). Thus, A62 mimics αCT binding while binding to L1 and bridges site-1′ (L1′) and site-2 (FnIII-1).

A mutational analysis confirmed that disrupting the αCT′–L1 interaction significantly enhances the potency of A62, whereas augmenting L1–αCT′ interaction significantly inhibits A62 activity^9^. Similar to the A62 aptamer, an insulin-mimetic peptide, S519, also competes with αCT′ and selectively activates the IR via m-pY1150^9,57^. This suggests that the displacement of αCT′ from L1 is an important step to induce m-pY1150 of IR. The structure of IR complexed with S597, another insulin mimetic, reveals that the IR forms an extended T-shape, whereby the two L1-CR-L2 arms are lifted upward with respect to the FnIII stalks; however, the structure is very similar to that of IR in the arrowhead conformation (Fig. 7a; discussed below)^17^. The phosphorylation state of S597-bound IR is unknown. However, because the S597 and S519 peptides harbor identical N- and C-terminal sequences, which bind FnIII-1 and L1’, respectively, it is reasonable to speculate that S597-bound IR is in the m-pY1150 state^17,57^.

## Tilted-T (*T*, asymmetric T or tow-crane)-shaped IR

Because multiple insulin molecules can bind to one IR, multiple IR conformations are possible. Negative staining of the nanodisc-trapped IR in the presence of different concentrations of insulin revealed several IR conformations^58^. One of these conformations appeared as a tilted T shape. High-resolution structures of tilted T-shaped IR have been identified under various conditions and can be classified into two functional classes.

In one type, the tilted T-shaped IR binds to one insulin and one A62 aptamer (Fig. 4g)^9^. In this complex, one half of IR (blue) is identical to the insulin-bound module of the Г-shaped IR, while the other half (orange) is identical to the arrowhead-shaped IR in which A62 is lodged between site-1′ (L1′) and site-2 (FnIII-1). In the head of the insulin-bound module, L1, CR, and L2 are lifted-up with a single insulin occupying site-1. In contrast, L1, CR, L2, and part of FnIII-1 are linearly arranged in an extended conformation in the A62-bound module. Notably, the membrane proximal ends between FnIII-3 and FnIII-3′ (37–45 Å apart) are similar to those in the arrowhead-shaped IR complexed with two A62 aptamers, even in the presence of insulin. Moreover, mutational analysis revealed that the tilted T-shaped IR is in the m-pY1150 state, similar to the arrowhead-shaped structure^9^. Therefore, we speculate that tilted T-shaped IR is one of the early intermediates between the inactive Λ-shaped and fully active Г-shaped IR. A62 likely traps the IR in an insulin-induced conformation via its interaction with the insulin-free protomer of the IR dimer.

**Fig. 5:**
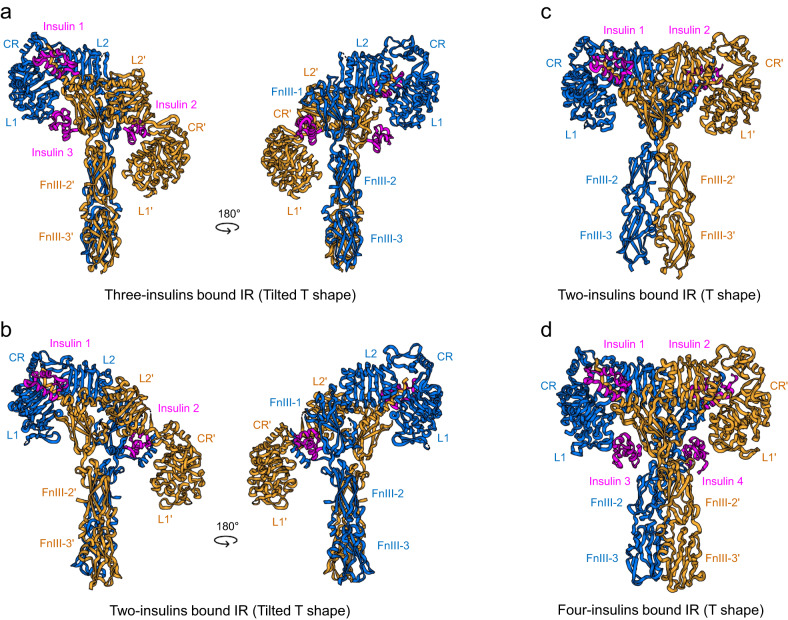
The cryo-EM structures of IR bound with two, three or four insulin molecules. The structure of tilted T-shaped IR complexed with three human–venom insulin hybrids (PDB ID: 7PG3) **a** with two insulin molecules (PDB ID: 7STK). **b** The cryo-EM structure of T-shaped IR bound to two insulin molecules (PDB ID: 8GUY) and **c** with three insulin molecules (PDB ID: 6SOF). **d** Insulin and insulin analogs are pink.

Other tilted T-shaped structures were observed in the presence of 2–3 insulin molecules, insulin mutants that cannot bind to site-2, and snail-venom insulin^15,19^. In an IR bound to three insulin molecules (PDB IDs: 7PG0, 7PG2, and 7PG3), the first insulin binds to the canonical insulin-binding site (site-1), the second insulin binds to FnIII-1′ at site-2’ on the same side of the IR dimer, and the third insulin simultaneously binds to site-1′ and site-2 in a noncanonical manner (Fig. 5a)^15,16,19^. The overall structure of two-insulin-bound IR is almost identical to that of three-insulin-bound IR, but without the an insulin molecule at site-2′ (Fig. 5b)^15^. Noncanonical insulin binding is achieved via its interactions with L1′ in one protomer and FnIII-1 in the opposite protomer, and this binding conformation is almost identical to the A62 bridge established between L1 and FnIII-1′ in arrowhead-shaped IR (Figs. 2f and 4d). Thus, the A62 bridge mimics site-1/site-2′ insulin coordination and traps IR in an intrinsically unstable noncanonical insulin-bound conformation.

These tilted T-structures were observed only in the presence of insulin at high concentrations that exceeded those of physiological conditions. The interactions of L1′ (on the insulin-free side) with FnIII-2 and FnIII-2′ are critical for stabilizing the distance between the membrane proximal ends (26 Å in Г-shaped IR). Thus, the distance between the membrane proximal ends in a tilted T-structure is likely to be similar to that in two-A62-bound IR. However, the A43-bound Γ-shaped IR structure showed that binding of a single insulin molecule to IR initiates complete IR phosphorylation without additional insulin molecules binding IR. Thus, the IR is likely fully phosphorylated in the second class tilted T-state, implying that the tilted T-conformations with two or three insulin molecules represent late intermediate confirmation between the single-insulin-bound Γ-shaped and four-insulin-bound T-shaped structures.

## T-shaped fully-active IR

IR forms a T-shaped conformation in the presence of two or four insulin molecules (Fig. 5c, d). The classification of the A43–IR complex in a dataset revealed that two insulin molecules can bind to site-1 and site-1′ when the insulin concentration was 200 nM^9^. At micromolar insulin concentrations, four insulin molecules independently occupied all insulin-binding sites (site-1, site-1′, site-2, and site-2′) of the T-shaped IR^12,13^. Thus, it is possible that a two-insulin-bound T-shaped IR is not very stable, and the binding of additional insulin molecules at site-2 and site-2′ stabilizes it. In multiple-insulin-bound conformation, the head forms a compact conformation whereby L1 is bent from the L1, CR, and L2 head axes and directed toward FnIII-2, and stalks are formed by the linear arrangement of FnIII-1, FnIII-2, and FnIII-3. The tips of the membrane-proximal ends of FnIII-3 are 36–40 Å apart.

An analysis of A62-bound structures revealed that the distance between the membrane-proximal ends of FnIII-3 determines the IR phosphorylation state. In the Γ-shaped IR structure, the FnIII-3 ends are 15–26 Å apart, which is the shortest in all reported structures. In contrast, in mono-phosphorylated IR structures (the arrowhead- and tilted T-shaped confirmations), the FnIII-3 ends are farther apart at distances of 34–45 Å. In the T-shaped conformation (two- or four-insulin-bound IR), this distance ranges from 36–40 Å, which is greater than that in Γ-shaped IR. Despite this distance, the T-state is reported to be fully activated^12^. Because single insulin molecule in the Γ-shaped IR conformation can induce the complete phosphorylation of multiple tyrosine residues in IRs at nanomolar concentrations, a conformational transition to a T-shaped state may be induced after a single insulin molecule binds, and further conformational changes may be induced by the binding of additional insulin molecules^9,56^. However, the function of the T-shaped conformation in downstream pathway activation is unclear, and its role in cellular functions requires further investigation.

## Conventional model for insulin binding and negative cooperativity

A hallmark of the insulin–IR interaction is its negative cooperativity; the binding of additional insulin molecules accelerates the dissociation of previously bound insulin^59,60^. However, the dose–response curves for the negative cooperativity of insulin binding are bell-shaped, indicating that the dissociation acceleration changes in a concentration-dependent manner. At insulin concentrations <100 nM, the dissociation rate of previously insulin is accelerated as the insulin concentration is increased. In contrast, the negative cooperativity gradually disappears as the insulin concentration is increased to be >100 nM. Moreover, Scatchard plots of insulin binding to holo-IR [(αβ)_2_ homodimers] are curvilinear, implying that IR carries separate high- and low-affinity sites for insulin binding. However, the Scatchard plot of insulin binding to the αβ monomer of IR was linear, corresponding to the low-affinity state^61,62^.

**Fig. 6:**
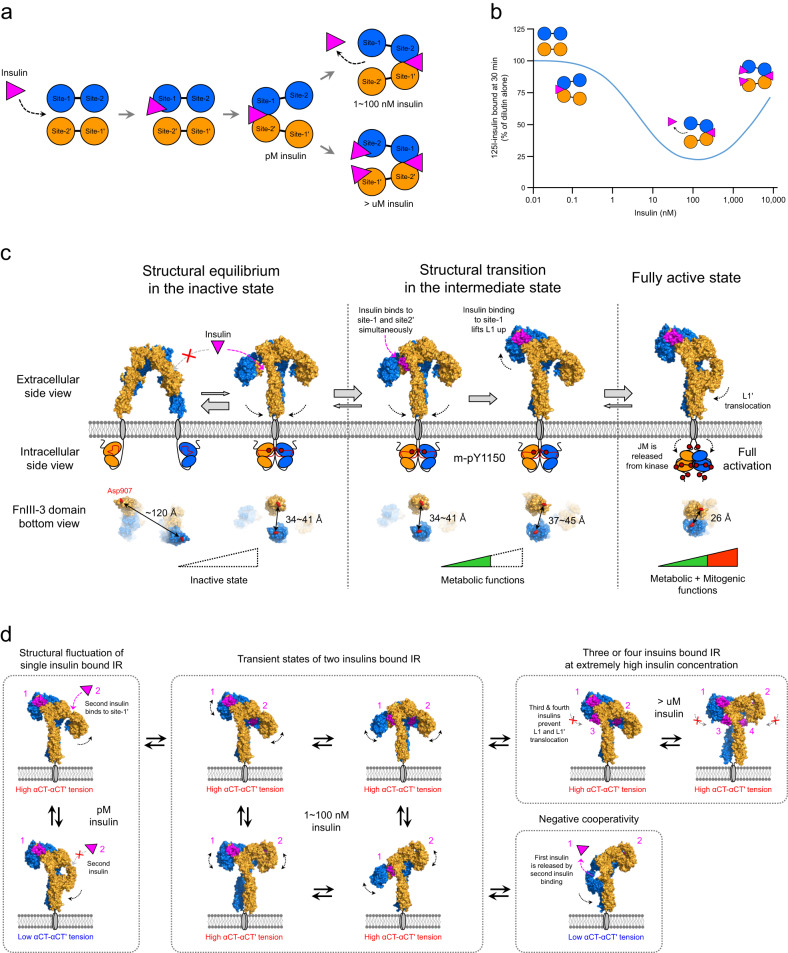
The proposed model for insulin-induced IR activation and negative cooperativity of insulin binding. **a** A conventional bivalent-crosslinking model showing the insulin binding mechanism^50,63^. At low insulin concentrations (~pM), the first insulin molecule binds strongly to the IR dimer by simultaneously interacting with site-1 and site-2’. As the insulin concentration increases (1–100 nM), the dissociation of previously bound insulin from IR is accelerated by a second insulin molecule binding to the opposite site-1’ and site-2 (negative cooperativity). At very high insulin concentrations (>μM), the binding of the second and third insulin molecule to each site-1’ and site-2 prevents the dissociation of the previously bound insulin from IR. **b** Dose‒response curve for the negative cooperativity of insulin binding to IR^59,60^. The bell-shaped curve indicates that accelerated dissociation of bound insulin changes in a concentration-dependent manner. **c** Stepwise activation model for insulin receptor activation from the extracellular and intracellular views. Insulin is pink, and arrows with dotted lines represent critical conformational changes in each step. The FnIII-3 domain bottom view represents the distance between the membrane proximal ends of FnIII-3 and FnIII-3′ in each step. **d** Model for the negative cooperativity of insulin binding. The dissociation of previously bound insulin is initiated by binding of a second insulin molecule to site-1’ and site-2. Two-insulin-bound IR undergoes transitions among various conformational states due to its intrinsic instability. When two insulin-bound IR structures transition into the Γ-shaped structure, previously bound insulin is dissociated from IR.

To explain these insulin-binding properties, a cross-linking model wherein each αβ monomer carries two different insulin-binding regions (site-1 and site-2) has been proposed (Fig. 6a)^50,51,63^. This model suggests that site-1 (or -1′) and site-2′ (or -2) are arranged in an antiparallel manner and that one insulin asymmetrically binds to site-1 with low affinity and then forms high-affinity cross-links by simultaneously binding to site-2′ on the other protomer. As the insulin concentration increases, a second insulin molecule binds to the opposite binding site pair (site-1′ and site-2) in the IR dimer, which accelerates the dissociation of the first-bound insulin. However, at insulin concentrations higher than ~100 nM, two additional insulin molecules independently occupy site-1′ and site-2, stabilizing the cross-link of the previously bound insulin, thus explaining the bell-shaped curve for negative cooperativity (Fig. 6b).

However, the single-insulin-bound Γ-shaped IR structure raises questions regarding conventional cross-linking models. First, one side of a single insulin molecule forms a stable complex with L1 and αCT′ (site-1); however, the other side of insulin, which interacts with site-2′, does not significantly interact with FnIII-1’. This structure is incompatible with the high-affinity cross-linking of insulin suggested by the conventional model. Second, the L1′ and αCT domains opposite the previously bound insulin module are located very close to FnIII-2 and FnIII-2′. To achieve effective negative cooperativity, the second insulin should be accessible to site-1’ (*K*_*d*_ 10–30 nM) in the single-insulin-bound IR. However, the empty site-1′ is not spatially exposed enough to allow binding of a second insulin molecules to IR in the Γ-shaped conformation. As FnIII-1 in the Γ-shaped IR structure is completely exposed, the second insulin may bind to site-2 first and induce subsequent site-1’/site-2 cross-linking after a transient conformational change, as suggested by Nielsen et al. ^16^. However, the *K*_*d*_ value of insulin binding to site-2’ (~400 nM) is greater than the concentration (1–100 nM) at which insulin exhibits marked negative cooperativity. Therefore, although the conventional cross-linking model has been proposed based on insulin-binding analyses, it cannot fully explain the negative cooperativity of the single-insulin-bound IR complex structure.

## Model for stepwise activation of IR

Although the arrowhead-shaped IR conformation is observed only when IR is bound to A62, it provides insights into insulin-binding-induced IR activation. A62 binding selectively induces m-pY1150 in the IR intracellular kinase domain^23^. Considering that Y1150 is the first tyrosine to be phosphorylated in the IR activation process, it is possible that the arrowhead-shaped conformation is a transient intermediate state between the inactive and fully activated IR. Moreover, the insulin bridge between site-1 and site-2′ in two-insulin-bound IR is similar to the bridge formed by A62^9^. These results suggest that the initial insulin binding occurs under site-1/site-2′ coordination in a manner similar to that of A62. Additionally, the crystal structures of partial IR domains (L1, CR, L2, FnIII-1, and αCT;5KQV) bound with insulin revealed a symmetric conformation almost identical to that of the head domain of A62-bound arrow-head-shaped conformation, supporting our model^21^.

However, in the Λ-shaped apo IR conformation, site-1 and site-2′ are in close proximity to each other, and the space between them is insufficient for A62 or insulin to form a bridge; this limits the possibility of insulin simultaneously binding to both sites when IR is in this conformation (Fig. 3g). One possibility is that site-2′ is the only initial insulin-binding site^16^. From a structural point of view, this assumption is feasible because the site-2′ interface in FnIII-1′ of the Λ-shaped conformation is completely exposed, and therefore, insulin can bind to it independent of site-1 involvement. Moreover, the T-shaped conformation in which four insulin molecules individually bind to one IR confirmed that a single insulin molecule can independently bind to site-2′. Thus, when insulin first binds to site-2′, site-1/site-2′ coordination may be induced by the insulin molecule that subsequently binds to site-1. However, how insulin binding to site-2′ of apo IR (Λ-shaped) induces subsequent conformational changes corresponding to IR activation remains unclear. Furthermore, site-2′-disrupted mutants showed partially reduced IR autophosphorylation and insulin binding, but mutations in site-1 residues completely disrupted IR autophosphorylation and insulin binding^9,12,49,64^. Notably, the T-shaped conformation wherein insulin binds to site-2′ is observed only when insulin is present at extremely high concentrations (uM). The binding affinity of insulin for site-2′ (*K*_d_ ~ 400 nM) is too low to initiate insulin binding at physiological insulin concentrations (pM)^50,51^. Therefore, we speculate that site-2′ plays an auxiliary role in site-1/site-2′ coordination in the early stage of IR activation.

Another possibility is that the arrowhead- and Λ-shaped conformations are in equilibrium in the absence of insulin (Fig. 6c). Consistent with this model, the Λ-shaped apo IR conformation was unstable and was resolved only in the presence of two types of antibodies that stabilized the structure^45,46^. Similarly, cryo-EM studies of the IR ECD confirmed that the structural states of apo IR are heterogeneous in the absence of insulin, making their structural analysis difficult^10,11^. Such structural flexibility can expose the L1 and αCT′ interface from FnIII-2′, allowing insulin to simultaneously access site-1 and site-2′. The protomer structure of the Λ-shaped IR is virtually identical to that of the arrowhead-shaped conformation (Fig. 4b). Thus, simple rigid-body rotation of each protomer can convert Λ-shaped IR to the arrowhead-shaped conformation.

According to our model, the initial binding of insulin to IR leads to transient arrowhead- and tilted T-shaped IR conformations, which are different from the fully active Γ-shaped conformation. An insulin molecule simultaneously binds to site-1 and site-2′, causing an equilibrium shift toward the arrowhead-shaped conformation. Because the binding affinity of insulin for site-2′ is significantly lower than that for site-1, site-1/site-2′ coordination is likely unstable and transient. Next, the insulin molecule dissociates from site-2′, which causes an upward shift of the insulin-bound site-1 module toward the upper loop of FnIII-1′, resulting in a tilted T-shaped conformation. The C-terminals of FnIII-3 and FnIII-3′ in these intermediate states are 34–45 Å apart, and only Y1150 is phosphorylated. Moreover, these intermediate IR structures display functional selectivity biased toward the PI3K/AKT pathway and metabolic functions (discussed below).

A hallmark of the fully-active Γ-shaped conformation is the binding between two αCT′/αCT helices and the L1/L1′ domains of IR, which are connected by a disulfide bond in the IDs (Fig. 3d). Thus, insulin binding lifts up L1 and αCT′, pulling the opposite L1′ and αCT toward the parallel FnIII-2/FnIII-2′ stalks, resulting in the transition to the fully active Γ-shaped conformation. This movement of the opposite L1′ brings the two FnIII-3 ends in close proximity, 26 Å apart; at this distance, complete phosphorylation of multiple tyrosine residues of IR is possible, thereby activating the MAPK pathway and mitogenic functions. However, the fact that A43 binding to IR dramatically markedly stabilizes insulin binding and increases IR autophosphorylation suggests that not all single-insulin-bound IRs stably form the Γ-shaped conformation^56^. Therefore, we anticipate that some proportion of IRs in peripheral tissues are fully activated by insulin, while others remain in partially activated intermediate states.

A key feature of the transition from the m-pY1150 to fully phosphorylated state is the decrease in distance between the FnIII-3 and FnIII-3′ ends (from 34–45 Å to 26 Å). In most RTKs, the distance between the kinases of the two monomeric receptors is a primary regulatory factor in receptor *trans*-autophosphorylation. The m-pY1150 of IR is also mainly initiated by a decrease in the distance between the cytoplasmic kinase domains. However, disrupting the interaction between JM and kinase domains by replacing Tyr972 with an Ala residue induced the phosphorylation of all the Tyr residues, even in the intermediate state^9^. These results imply that, in addition to the distance between the two kinases, the subsequent conformational rearrangement of the intracellular IR domains is important to determine the IR phosphorylation state.

A limitation of our model is that it does not explain how a small difference in the distance between the FnIII-3 and FnIII-3′ ends regulates the interaction between JM and kinase domains, which in turn results in the distinct phosphorylation states of IR. As phosphorylation of all the tyrosine residues in IR occurs in *trans*, agonist bias toward m-pY1150 indicates that two IR kinases are in sufficient proximity to induce *trans*-autophosphorylation even when the two FnIII-3 ends are separated by 34–45 Å. However, at this distance, phosphorylation of Y1146 and Y1151, which are located near Y1150, is restricted. To date, structural evidence to explain how these intracellular rearrangements regulate the substrate specificity of IR kinases for each tyrosine residue is lacking. Therefore, a structural analysis of full-length IR in the basal, intermediate, and fully active states is essential for a deeper understanding.

## Proposed mechanism for the negative cooperativity of insulin binding to IR

Although up to four insulin molecules bind to one IR in 1:1 to 4:1 stoichiometry, depending on the insulin concentration, the physiological role of multiple insulin molecules binding to IR remains unclear. Interestingly, the A43 aptamer can fully saturate IR phosphorylation even at low insulin concentrations by stabilizing the single-insulin-bound Γ-shaped conformation. Moreover, A43 binding disrupts the second and third insulin-binding sites by masking site-1′ and site-2. Thus, the A43-bound IR structure suggests that IR can be fully activated in the Γ-shaped conformation without additional insulin binding. The blood insulin level ranges from ~50 pM (after overnight fasting) to ~500 pM (after meals) under normal physiological conditions^65^. Local insulin concentrations (1–5 nM) in the liver and pancreas are higher than circulating insulin concentrations because insulin secreted from the pancreas is directly delivered to the liver through the hepatic portal vein^66,67^. However, physiological insulin concentrations are significantly lower than the concentrations at which multiple insulin molecules bind one IR (>100 nM). Therefore, it is reasonable to speculate that multiple insulin molecule binding to IR is unlikely to occur in most peripheral tissues, and 2–4-insulin-bound IR structures are observed only in the presence of insulin at excessive concentrations, not under normal physiological conditions.

Nevertheless, the multiple-insulin-bound IR structures provide important insights into the negative cooperativity of the insulin–IR interaction. The two insulin-bound tilted-T-shaped IR conformation explains the mechanism through which a second insulin molecules binds to IR in the Γ-shaped conformation (Fig. 6d). The position of L1′ opposite insulin bound to IR in the Γ-shaped conformation may be unstable, allowing a second insulin to establish site-1′/site-2 coordination. Because αCT and αCT′ are cross-linked via a Cys683–Cys683′ disulfide bond, the position of L1′ can cause steric tension between L1 and L1′. Thus, the binding of a second insulin molecule forces a downward shift of the first insulin-bound L1. As the force of steric tension caused by the αCT–αCT′ bridge and the force reverting IR to the stable Γ-shaped conformation counteract, two-insulin-bound tilted T-, arrowhead-, and T-shaped IR conformations may be transient and undergo rapid switching from one conformation to another.

Despite these structural fluctuations, IR is eventually converted to the most stable conformation (Γ-shaped IR), and one of the two bound insulin molecules dissociates from it. As the insulin concentration increases, the opportunity for a second insulin to bind to IR increases, and negative cooperativity, which accelerates the dissociation of previously bound insulin, also increases. However, at high insulin concentrations (>100 nM), binding of a third and fourth insulin molecules to site-2 and site-2′ begins to stabilize the tilted T- and T-shaped conformations by preventing the translocation of L1 or L1′. Thus, the binding of the third and fourth insulin molecules prevents the dissociation of the previously bound insulin, leading to attenuated dissociation acceleration.

## Functional selectivity of IR

Over the past few decades, several artificial ligands targeting IR have been developed based on small molecules, peptides, antibodies, and aptamers^22,23,25,56,68–72^. Although insulin functions as both a metabolic regulator and growth factor, some artificial IR-targeting agonists have been identified as biased agonists that lead to the functional selectivity of intracellular signaling and cellular function activation. Functional selectivity (also called “biased agonism”) describes the ability of ligands to selectively activate specific signal pathways and induce precise cellular functions compared to other ligands that can fully activate the same receptor^73^. In the case of IR, biased agonists show properties similar to those of other ligands, normally activating the PI3K/AKT pathway, but they do not affect the MAPK pathway. Consistent with selective signaling pathway activation, biased IR agonists strongly stimulate metabolic functions, such as glucose uptake and glycogen synthesis, but exert little or no effect on cell proliferation^74^. Studies on two agonist aptamers, IR-A48 and IR-A62, confirmed that these functionally selective aptamers phosphorylate only Y1150 (m-pY1150) in the IR kinase activation loop^22,23^.

**Fig. 7:**
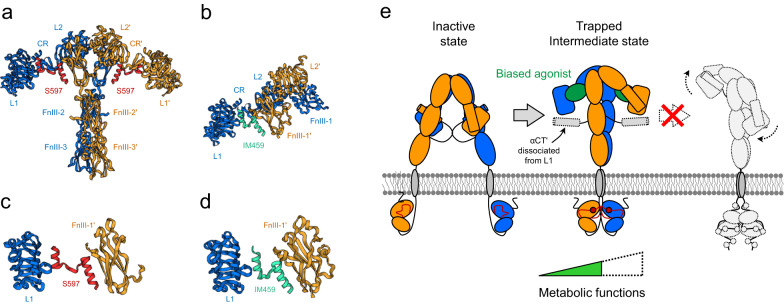
Functional selectivity of IR. **a** The cryo-EM structure of extended T-shaped IR bound to two S597 (PDB ID: 8DTL). **b** The cryo-EM structure of partial domains of IR bound to IM459 (PDB ID: 7U6D). Detailed binding mode of S597 (**c**) and IM459 (**d**). Both peptides bind to site-1 and site-2’ simultaneously, similar to the A62 in the arrowhead-shaped IR, which forms a bridge. **e** Proposed model of the functional selectivity of IR. Cross-linking between L1 and FnIII-1’ with the dissociation of αCT′ from L1 establishes the intermediate state of IR, which may be critical for its metabolic effects.

Biased agonist peptide- or aptamer-complexed IR structures exhibit a few common features that provide clues to the function of selective agonists (Fig. 7a, b). The peptide agonist S597 binds to IR to form an extended T-shaped conformation (8DTL) similar to the two-A62-aptamer-bound arrowhead-shaped IR conformation (Fig. 7a)^17^. Similar to that in the arrowhead structure, the L1, CR, L2, and part of FnIII-1 are arranged in a linear and extended conformation in a symmetrical manner. However, the head is further lifted compared to its position in the arrowhead-shaped conformation, resulting in the formation of an extended-T shape.

Similar to the A62 aptamer, S597 simultaneously interacts with L1′ and FnIII-1 in IR (Fig. 7c). Another biased peptide agonist, IM459, also cross-links with L1′ and FnIII-1 (7U6D, Fig. 7b, d)^14^. Moreover, the IR residues that interact with the S597 peptide are identical to or in close proximity to those in the A62-bound IR at site-1/site-2′; R14, L62, F64, R65, and F96 in L1 and Y477, R479, L486, R488, W553, and L554 in FnIII-1′ are involved in selective agonist binding. These findings suggest that the functional selectivity of IR is related to the intermediate states of the receptor. In both structures, αCT is liberated and disordered from L1 by A62 or S597, preventing the intermediate structures (arrowhead- and T-shaped) from transitioning to the fully active Γ-shaped structure. This is because F64 and F96 are critical for the interaction between αCT′ and L1, which plays an important role in the insulin binding to site-1.

Two features of IR provide important clues for the design of ligands that selectively activate IR (Fig. 7e). The first one is the displacement of αCT’ from the L1 surface. Although it does not interact with FnIII-1’, the short C-terminal sequence of S519 (S519-C16; 16 residues) induces m-pY1150, thereby demonstrating that the structural disruption of the inactive Λ-shaped structure is sufficient to induce a transition to intermediate IR states^9,57^. Moreover, structural and biochemical studies confirmed that E697 and E706 in the N-terminal portion of αCT’ bound to L1 interact with L2 and FnIII-1’, respectively, and play a critical role in the formation of a fully active Γ-shaped structure and normal insulin action^12,48,49^. If some compounds can crosslink L1 and FnIII-1’ without αCT’ displacement, such as an insulin bridge between site-1 and site-2’ (Fig. 2f), theoretically, they can induce m-pY1150 to activate IR. However, these compounds are expected to be highly inefficient because they must compete with the normal action of insulin, which strongly binds to site-1. Thus, the displacement of αCT’ from L1 is an effective way to prevent intermediate structures from transitioning to the fully active Γ-shaped structure. The second important feature is the cross-linking between L1 and FnIII-1’. Full-length S519 containing the N-terminal sequence that interacts with FnIII-1’ showed an effect similar to that of S519-C16 (2 mM) by inducing m-pY1150 at a 100-fold lower concentration (20 μM)^9^. Cross-linking L1 and FnIII-1’ seems to markedly enhance the potency of S519 by stabilizing the intermediate structures of IR.

Our model for stepwise IR activation is useful for describing the relationship between the conformational states and functional selectivity of IR. To date, IR phosphorylation is recognized as a continuous step in its transition from an inactive to a fully active state. However, the insulin mimetic peptides S519, A48, and A62 selectively induced m-pY1150 in the IR kinase domain by stabilizing an intermediate IR structure^9^. Considering that these biased agonists selectively initiate the PI3K/AKT pathway and exert metabolic effects, the phosphorylation state and their functional selectivity of IR appear to be related.

However, whether m-pY1150 is a critical factor in determining intracellular signaling pathways is unclear. Although Y1150 plays an important role in regulating IR kinase activity, kinase activity alone does not determine the direction of intracellular signaling pathways. Signaling outcomes resulting from receptor activation are influenced by various factors, such as tissue type, cytoplasmic location of the receptor, receptor endocytosis, ligand-binding dynamics, and dimer stability^75–78^. The two major IR pathways also differ in the mechanism by which signaling is initiated by activated IR. For instance, although both IRS-1 and SHC have a phosphotyrosine-binding domain that binds to phosphorylated Y960 (pY960) in the IR JM domain, their dependence on pY960 for triggering signaling differs^38,79^. Phosphotyrosine-binding domain deletion studies demonstrated that the insulin-induced phosphorylation of SHC is mediated completely by pY960 in IR, but the phosphorylation of IRS-1 was only partially decreased. Thus, IR functional selectivity is related to its interaction with various adapter proteins; however, how the phosphorylation state of the receptor determines this selectivity requires further study.

In contrast to insulin, which results in fast IR internalization, S597 did not induce IR internalization, which suggests a plausible mechanism underlying IR selectivity toward PI3K/AKT pathway activation^24^. The PI3K/AKT pathway is rarely affected by IR internalization; however, full activation of the MAPK pathway essentially requires insulin-induced IR internalization^80–82^. As discussed in the previous section, A62 and S597 simultaneously interact with L1’ and FnIII-1 in IR. S597 is an S519 analog that shares the exact same N- and C-terminals sequence, which are needed for FnIII-1 and L1’ binding^17^. Thus, similar to A62 and S519, S597 is likely to selectively induce m-pY1150. pY960 at the intracellular NPXY motif in IR plays an essential role in insulin-induced IR internalization. The deletion of NPEY^960^ or substitution of Y960 with a phenylalanine residue (Y960F) considerably decreased the insulin-induced IR internalization^83,84^. Thus, these studies together with the structural evidence support the idea that m-pY1150 of IR induced by biased agonists may lead to impaired IR internalization due to the lack of pY960, resulting in the selective activation of PI3K/AKT pathway. Further studies are required to map the site-specific Tyr phosphorylation of IR by other biased agonists and investigate the effect of m-pY1150 on IR internalization.

## Therapeutic benefits of the IR intermediate state

The activation of inappropriate signaling by drugs that target receptors can cause a variety of adverse effects. However, it is difficult to resolve these problems when the drug is a full agonist that activates all the signaling pathways initiated by the receptor. In some cases, biased agonists can be better therapeutic agents because they selectively activate the signaling pathway required for treating a particular disease without inappropriate signaling activation^73,85^. Many biased agonists for G-protein coupled receptors (GPCRs) are being studied to evaluate their suitability in drug development, and the first GPCR-biased agonist drug, the μ-opioid agonist oliceridine, was approved by the USA Food and Drug Association (FDA) in 2020^86^.

In past decades, insulin has been the most effective and successful therapeutic agent for improving glycemic control in patients with diabetes^87^. Because type 2 diabetes (T2D) is intimately linked to chronic insulin resistance, patients with severe T2D commonly require a higher insulin dose than normal individuals. Some patients with type 1 diabetes (T1D), an autoimmune disease that leads to absolute insulin deficiency, eventually become insulin resistant because of the continuous administration of exogenous insulin. Therefore, these patients experience chronic exposure to high insulin doses, either endogenously produced or exogenously administered, for decades, which contributes to excessive stimulation of mitogenesis^8^.

The mitogenic stimulation induced by excessive insulin levels has been suggested to be a potential cause of diabetic complications and cancer progression^8,88–90^. Intensive glycemic control in patients with diabetes dramatically decreases the risk of microvascular complications, such as retinopathy, nephropathy, and neuropathy, but does not clearly prevent macrovascular complications, including myocardial infarction, stroke, peripheral vascular disease, and diabetic foot^91,92^. Atherosclerosis, caused by the development of an atheroma in the inner layer of arterial walls, is a major driver of macrovascular complications^7^. Normal insulin signaling stimulates the expression of endothelial nitric oxide synthase through PI3K/AKT pathway activation^93,94^. Nitric oxide secreted from the endothelium regulates the adhesion of white blood cells to vessels and vascular smooth muscle contraction, thereby preventing atherosclerosis^95^. However, under diabetic conditions, impaired PI3K/AKT pathway due to insulin resistance reduces nitric oxide production, while excessive activation of the MAPK pathway facilitates the progression of atherosclerotic lesions by increasing collagen synthesis and the proliferation of vascular smooth muscle cells^96–98^. Furthermore, increasing evidence suggests that obesity and diabetes are risk factors for several cancers^8,99^. The risks for cancerogenesis and cancer progression in patients with obesity and/or diabetes are associated with various systemic dysfunctions. Many cohort and case‒control studies have reported that hyperinsulinemia contributes to cancer risk^8^. Moreover, in vitro and in vivo studies have demonstrated that insulin and IR are strongly associated with cancer progression^100–103^. The causal relationships between hyperinsulinemia and cancer are complicated and vary on a case-by-case basis but are believed to be caused by excessive stimulation of cell proliferation and growth by insulin and IR.

As lowering blood glucose is the most urgent goal for patients with diabetes, potential adverse effects of long-term insulin administration or hyperinsulinemia are generally ignored. IR inactivation using inhibitors or caloric restriction to lower blood glucose levels can be used clinically to reduce circulating insulin levels; however, these strategies are practically infeasible for patients with severe diabetes who need exogenous insulin administration. Theoretically, biased agonists that maintain an IR conformation in the intermediate stage may be an alternative to resolve this ironic problem. Because biased agonists can induce glucose uptake through selective activation of the PI3K/AKT pathway without mitogenic stimulation, they show the potential to alleviate the adverse effects of insulin administration or hyperinsulinemia. However, no in vivo studies on the effects of biased agonists on cancer progression in animal models of diabetes have been reported. In 2018, only one study reported that the insulin mimetic peptide S597, a biased agonist, retarded atherosclerotic lesion progression, which was achieved through a process associated with protection from leukocytosis, in Ldlr-/- mice^104^.

## Conclusion and future perspectives

Functional selectivity induced by different ligands has been observed in RTKs in addition to IR, including the fibroblast growth factor receptor, ErbB (epidermal growth factor) receptor family, platelet-derived growth factor receptor family, RET (rearranged during transfection) receptor, Ephrin receptor family, and TRK (tropomyosin receptor kinase) receptor family^76^. For instance, the ErbB family, consisting of four RTKs (ErbB1–4), interacts with 11 types of endogenous ligands, and these ligands lead to different receptor phosphorylation states and functional outcomes *via* the same receptor^105^.

To date, the activation mechanisms of these RTKs have been explained as simple on/off transitions between inactive (unliganded) and active (liganded) states. Most RTKs, including IR, share highly conserved mechanisms for receptor activation, such as ligand-induced receptor dimerization, a decrease in the distance between two kinases, *trans*-autophosphorylation, tyrosine phosphorylation in a kinase activation loop, and the interaction between JM and the kinase^28,75,78^. Thus, it is plausible that the activation of other RTKs also involves stepwise process and that different rearrangements of intracellular domains induced by different ligands may be involved in functional selectivity. The mechanism of the functional selectivity of the IR explained herein can be generalized to that of other RTKs.

One of the major reasons for the lack of studies on the selective activation of IR thus far is that a clear mechanism has not yet been elucidated at the receptor level. Therefore, the rational design of biased agonists that can be applied to in vivo experiments has been impossible. However, recent advances in the structural studies of IR–ligand complexes have provided a reasonable basis for the design of functionally selective IR agonists. Thus, the development of stable and effective biased agonists with potential clinical applications is one of the most important goals of future studies. We hope that studies on biased agonists of IR will provide opportunities to further advance the treatment of diabetes.
